# Real Time Monitoring of Wine Vinegar Supply Chain through MOX Sensors

**DOI:** 10.3390/s22166247

**Published:** 2022-08-19

**Authors:** Dario Genzardi, Giuseppe Greco, Estefanía Núñez-Carmona, Veronica Sberveglieri

**Affiliations:** 1 National Research Council, Institute of Bioscience and Bioresources (CNR-IBBR), Via J.F. Kennedy, 17/i, 42124 Reggio Emilia, RE, Italy; 2 Nano Sensor Systems S.r.l., (NASYS) Spin-Off University of Brescia, Via Camillo Brozzoni, 9, 25125 Brescia, BR, Italy

**Keywords:** MOX sensors, wine vinegar, real time analysis, pied de cuve

## Abstract

Vinegar is a fermented product that is appreciated world-wide. It can be obtained from different kinds of matrices. Specifically, it is a solution of acetic acid produced by a two stage fermentation process. The first is an alcoholic fermentation, where the sugars are converted in ethanol and lower metabolites by the yeast action, generally *Saccharomyces cerevisiae*. This was performed through a technique that is expanding more and more, the so-called “pied de cuve”. The second step is an acetic fermentation where acetic acid bacteria (AAB) action causes the conversion of ethanol into acetic acid. Overall, the aim of this research is to follow wine vinegar production step by step through the volatiloma analysis by metal oxide semiconductor MOX sensors developed by Nano Sensor Systems S.r.l. This work is based on wine vinegar monitored from the grape must to the formed vinegar. The monitoring lasted 4 months and the analyses were carried out with a new generation of Electronic Nose (EN) engineered by Nano Sensor Systems S.r.l., called Small Sensor Systems Plus (S3+), equipped with an array of six gas MOX sensors with different sensing layers each. In particular, real-time monitoring made it possible to follow and to differentiate each step of the vinegar production. The principal component analysis (PCA) method was the statistical multivariate analysis utilized to process the dataset obtained from the sensors. A closer look to PCA graphs affirms how the sensors were able to cluster the production steps in a chronologically correct manner.

## 1. Introduction

The name vinegar is reserved for the product obtained exclusively by the biological process of double fermentation, alcoholic and acetous, from liquids or other substances of agricultural origin [1]. Vinegar is a fluid with a low pH obtained by the acetic fermentation of alcohol (and/or carbohydrates) due to the action of aerobic bacteria. The most widely used and productive strains are those of the genus Acetobacter. Specifically, acetic acid bacteria AAB are classified in the following genera: *Acetobacter, Gluconobacter*, *Gluconacetobacter*, *Acidomonas*, *Asaia*, *Kozakia*, *Swaminathania*, and *Saccharibacter* [2]. Vinegar contains approximately 5% acetic acid in water, varying amounts of fixed fruit acids, coloring matter, salts, and a few other fermentation products that impart characteristic flavor and aroma to the product [3]. Vinegar is a liquid composed mainly of water, acetic acid, alcohol, aldehydes, and compound ethers; in dilution, there are also free amino acids and mineral salts. Vinegar does not evaporate and freeze identically to water. While the evaporation point of its water portion is approximately 100 °C, that of acetic acid is considerably higher, approximately 120 °C. In addition, unlike water, which reaches a solid consistency at approximately 0 °C, acetic acid has a freezing temperature of approximately −17 °C. Natural vinegars, as they come from the generators, normally contain an excess of 4 g of acetic acid per 100 mL. When vinegar is diluted with water, the label must bear a statement, such as “diluted with water to XXX percent acid strength”, with the blank filled with the actual percentage of acetic acid in no case should it be less than 4% [4]. However, food vinegar has a percentage of acetic acid that generally fluctuates between 4.5% and 12% (depending on the type), which is why it freezes and evaporates more similar to water as a whole than pure acetic acid [5]. Vinegar is produced in barrels, tanks, or autoclaves in which the wine and the specific organic starter are placed. Inside, the compound is continuously aerated because the microorganisms responsible for fermentation are of the obligated aerobic type. The alcohol content of the initial liquid should be between 8 and 10% (since the actual yield of the transformation is approximately one gram of acetic acid per gram of alcohol), while the optimal temperature is approximately 25–30 °C [6]. The dilution plays a fundamental role in the reaction of some elements, such as phosphorus, calcium, iron, and manganese. As a consequence, these conditions allow for the microbiological development and formation of the so-called mycoderma vinegars, a surface layer of bacteria and cellulose-like organic substances. Vinegar must also be filtered to remove mycoderma suspended vinegars before the commercialization. Therefore, vinegar can be produced from any sugar source convertible in an alcoholic matrix [7]. AAB more utilized are members of the genus Acetobacter and characterized by their ability to convert ethyl alcohol (CH_3_CH_2_OH) into acetic acid (CH_3_COOH) by oxidation. Wine vinegars must contain an acetic acid concentration between 6 and 12% and a maximum ethanol concentration of 1.5%. Vinegar production must be carefully checked step by step. One of the critical steps in vinegar production is the preparation of the raw material [8]. The correct acidification of the fermentable sugar and juice solution shall be obtained through this step. The processing differs depending on the raw material used. In general, fruits require less preparation than seeds; however, seeds are more easily stored and preserved after harvest. Fruits are highly perishable, rich in water, and need to be processed very quickly. Therefore, basic safe food handling practices, storage, and processing are essential to prevent the growth of pathogenic microorganisms. These microorganisms could alter the quality of the final product or even produce dangerous toxins, such as aflatoxin [9]. The main fermentation systems to produce vinegar are two: liquid state fermentation and solid state fermentation [10].

Consequently, These Two Can Be Classified in Other Subclasses [11]

Liquid state fermentation can be consequently classified in:

1. Superficial or static fermentation (SFC): The obtained wine is maintained in an open-air container. Lactic acid bacteria (LAB) development will mainly be on the liquid surface, promoting the formation of a strong biofilm;
2. Submerged (SMR) fermentation: The entire liquid is subjected to an addition of air, agitated/mixed with self-priming turbines to increase the surface of contact with air.

Solid state fermentation (SSD) is based on the utilization of a solid support as:

1. carbon source/energy;
2. inert support;
3. carbon source/energy and inert support [12].

Currently, the classic techniques to follow vinegar production are several. Some examples are:

1. analysis of alcohol content;
2. pH analysis;
3. optical control;
4. acid-base titration.

All these techniques require sampling and strong training, and they are time consuming and unable to assure a real time monitoring. An excellent alternative is sensor analysis. Specifically, in this study semiconductor metal oxide sensors [13] (MOX) were used. This technique is based on the analysis of the volatile fingerprint of the matrix and is extensively explored, with the advantages of low cost, facile synthesis process, high sensitivity, quick responses, and favorable stability [14,15]. This approach has already been performed for the monitoring and the classification of several matrices, such as coffee, jam, or Extra Virgin Olive Oil (EVOO). Overall, the aim of this research is to investigate a new kind of approach, which would assure a real time monitoring of the vinegar supply chain. In addition, utilizing this technique would ensure a consistent work saving. Finally, the analysis would be entirely nondestructive, following step by step the wine vinegar production directly from the product, without the need of sampling.

## 2. Materials and Methods

### 2.1. Sample Preparation

**Figure 1:**
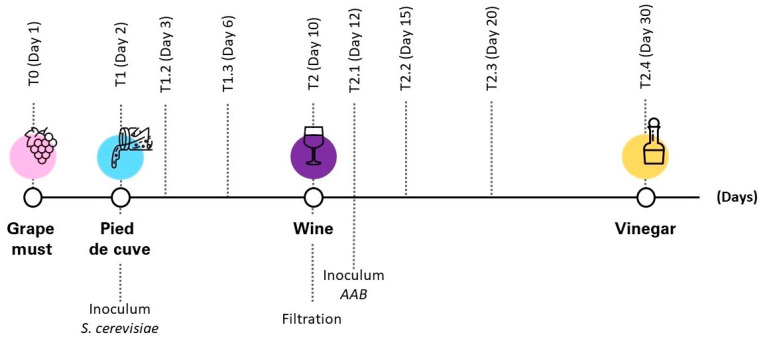
Flow sheet wine vinegar production.

This study is based on the analysis of wine vinegar. For the preparation of the vinegar, 5 L of concentrated grape must was inserted into a glass demijohn. The demijohn was placed inside a conditioned tank containing water, with a temperature set to 25 °C [16]. Conditioning at a controlled temperature is necessary to avoid the growth of alterative and/or pathogenic microorganisms. The upper part of the demijohn was then covered with an odorless film that allowed an exchange of oxygen with the external environment. Oxygen is fundamental to promoting the ethanol (CH_3_CH_2_OH) oxidation to acetic acid (CH_3_COOH) [17]. To give way to the first phase of alcoholic fermentation, we proceeded through the technique of “pied de cuve” with yeast *Saccharomyces cerevisiae* [18]; without this ancient technique the natural alcoholic fermentation would be left to chance, with many risks, such as fermentation of some non-Saccharomyces yeasts, which could create olfactory deviations [19]. This technique is based on the inoculation of a given amount of must, previously subjected to a partial alcoholic fermentation, into a new must. When the fermented pied de cuve reaches an ethanol concentration of approximately 5% (*v*/*v*), the pied de cuve is added to the must with a pied de cuve/new must ratio of 1:10 [20]. Thus, the pied de cuve method applies viable yeast cells to start a new fermentation and promotes the growth of yeasts with good fermentation characteristics [21]. In order to evaluate the progress of the first phase of alcoholic fermentation, the degree brix of the must was measured step by step, using the RF40-ND FLIR Extech refractometer. After settling to the value of 8° brix on the tenth day, the phase of alcoholic fermentation was considered finished. At the end of the alcoholic fermentation, the resulting wine was filtered using a paper filter with a diameter of 40 cm, and poured into a new clean glass demijohn. On the twelfth day, we proceeded by inoculating the wine obtained with a starter culture of Acetobacter vinegars, in order to start the acetic fermentation phase. The latter, compared to alcoholic fermentation, presents different problems and requires attention in the start-up phase [22]. By inoculating the starter culture, it is possible to start a controlled fermentation. For the final production of wine vinegar, we opted for the static fermentation system. In this way the hole of the glass demijohn was left open, covered only with an inert breathable film, so that the acetic bacteria attract the oxygen necessary for the transformation [23]. Below is a pictorial representation of the vinegar production process, the timeline associated with each step and the analysis time for each point measurement in days (Figure 1).

### 2.2. Small Sensor System (S3)

#### 2.2.1. Analysis Conditions

**Figure 2:**
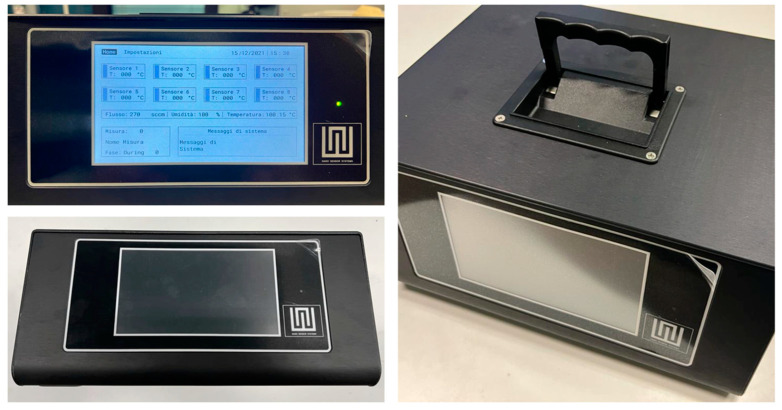
Small Sensory systems—S3+.

The S3+ device (Figure 2), acronym for Small Sensor Systems, is constructed by Nano Sensor Systems S.r.l. Reggio Emilia, Italy, www.nasys.it (accessed on 18 February 2022). This S3+ device is able to detect volatile compounds which are transported by a pump inside the device from samples.

The system allows for the collection and analysis of the data acquired in the cloud, making S3+ an IoT device for the management and control of signals. The sensor response is based on the change of its resistance over time caused by interactions with different kinds of volatile compounds or surrounding environments. The reactions between the oxygen species adsorbed on the surface of the sensor and the target molecules lead to the variation in the concentration of charge carriers in the sensing material, affecting its electrical conductance. This device has been already used with remarkable success in other previous studies in the field of quality control and food technology [24]. By using polyurethane pipes inserted inside the glass demijohn, the S3+ device is able to monitor the evolution of the sample continuously. The system allows for the collection and analysis of the data acquired in the cloud, making S3+ an IoT device for the management and control of signals [25]. S3 is composed of three essential parts [24]:

1. The steel chamber of the sensor contains the six MOX sensors. This allows the sensor to be separated from the environment, except for an inlet and an outlet path for the passage of volatile compounds. Other types of sensors are placed in order to control several parameters during the analysis. These are the temperature, humidity, and flow in the chamber;
2. The dynamic fluid circuit consists of a pump (Knf, model: NMP05B), polyurethane tubes, an electro valve, and a metal cylinder, which contains activated carbon. Activated carbon plays the filtering role for any type of odor in the environment that may alter the final response. The solenoid valve is positioned at the inlet of the chamber to control the flow of the pump, with a maximum of 250 sccm;
3. The electronic board processes the sensor responses by detecting the electrical resistance. It also controls the operating temperature of the sensors, which is an important parameter for the detection of volatile compounds. Finally, the system is able to send data to the Web App dedicated to the S3 device via an internet connection.

**Figure 3:**
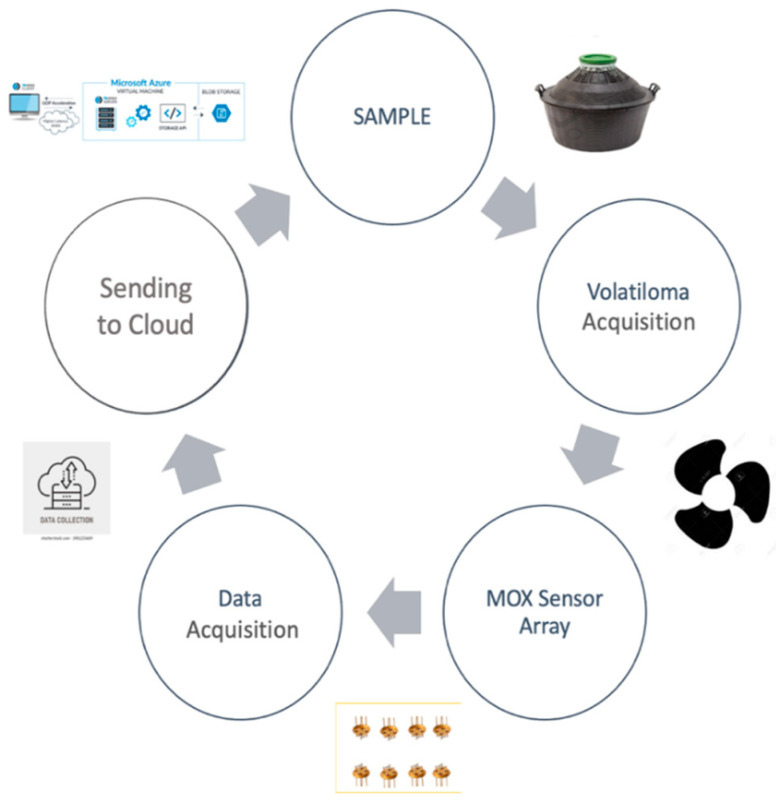
Flow Sheet Sensor Analysis.

Figure 3 shows the entire set up of the sensor analysis utilized in this work. The sensor response is based on the change of its resistance over time caused by interaction with different kinds of volatile compounds or surrounding environments.

The reactions between the oxygen species adsorbed on the surface of the sensor and the target molecules lead to the variation in the concentration of charge carriers in the sensing material, affecting its electrical conductance [14]. This EN has been successfully used in several applications, such as microorganism detection on food [26], characterization of EVOO [14] and coffee blends [27], or jam recipe identification [28].

#### 2.2.2. S3 Data Analysis

Processing of the S3 sensor signals was performed using MATLAB^®^ R2019b software (MathWorks, Natick, MA, USA) in order to extract the features of the sensor response. Sensors’ responses in terms of resistance (Ω) were normalized to the first value of the acquisition (R0). For all of the sensors, the difference between the first value and the minimum value during the analysis time was calculated. Hence, the ∆R/R0 parameter was calculated and has been used as a feature for all the sensors responses to the 36 replicates of each sample. The standard deviation of the ∆R/R0 parameter was calculated for each group of sample measurements prior to proceeding with PCA analysis, revealing a maximum uncertainty of the 10%. Once the data matrix was calculated, principal component analysis (PCA) was applied to the data in order to verify the variation of the volatile organic compounds (VOCs) in the different samples [29]. This technique consists of clustering the sample variables through linear combinations that describe the link between one sample to the others. This results in the main components (PC), which are far fewer than the original variables. These new variables are structured in such a way as to be orthogonal to each other (not correlated). Moreover, most of the variability of the samples is present in the first main components. As a consequence, PC1 shows the largest variation. Next, PC2 represents the second largest variation. This can continue until all the variables are explained. These conditions allow for the detection of any groupings [30]. They are also known as clusters that represent samples united by characteristics. PCA is not a classification technique, but a technique that may provide the distribution of the samples within the main components considered in the hyperplane.

## 3. Results and Discussion

**Figure 4:**
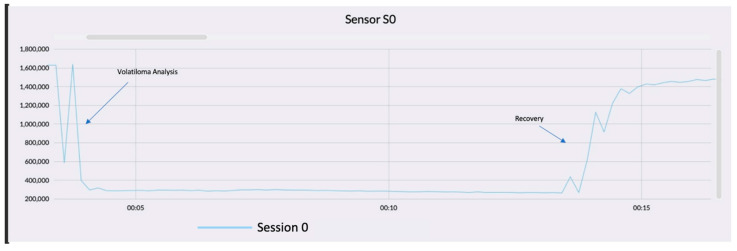
Typical sensor response (*y*-axis presents electrical resistance (Ohm), *x*-axis time shows in minutes.

Figure 4 shows the graph model of sensor response, where on the x-axis is indicated the time expressed in minutes, while the y-axis reports the normalized resistance. The use of normalized parameters allows for work on a dataset without dimensions, hence they are without a unit of measurement, where the variability is equal to 1 and the average is 0. Using normalized variables is advantageous in the analysis of samples that have different units of measurement or size, which would prevent accurate analysis of samples [27].

In the curve shown in the graph, it is clearly noticeable how the trend starts from a higher resistance value to one that is far smaller. The analysis of samples follows two phases: the first consists in the analysis of the sample volatiloma, which corresponds to the fall of the trend and where the chemicals volatilize up to contact with the sensor. Thus, this causes a decrease in the electrical resistance.

The second stage is called recovery. In this case, filtered ambient air is passed through, so that the sensor returns to its baseline. The latter is the electrical sensor resistance in air under standardized conditions (moisture, temperature, oxygen). In our case the first phase lasted 15 min, followed by the recovery to the base-line, which required 120 min, with a consequent total analysis time of 135 min.

Vinegar quality [31] and acceptance by consumers depends on several parameters, aroma being one of the most important. In wine-based vinegars and derived products as in all the other matrices, the volatiloma is the set of all volatile metabolites as well as other volatile organic and inorganic compounds that originate from an organism, super-organism, or ecosystem.

While all volatile metabolites in the volatiloma can be considered as a subset of the metabolome, it also contains exogenous derivative compounds that are not produced from metabolic processes (e.g., environmental contaminants), therefore volatiloma can be considered as a distinct entity from the metabolome. In this case, the volatiloma is the result of the contribution of several hundred volatile organic compounds (VOCs) belonging to different classes (e.g., mono- and sesquiterpenes, esters, higher alcohols, carbonyl, and sulphur compounds), encompassing a wide range of volatilities and polarities [32].

These VOCs may come from the raw materials (e.g., red wines, fruits, cider, malted barley, honey, among others) and/or may be formed during production and storage processes [33]. VOCs are present, to a large extent, in fruits and aromatic/medicinal herbs positively influencing the final quality of vinegars by the addition of new compounds derived from them [34]. In addition, fruits are rich in vitamins, minerals, and phytochemicals and contain potentially bioactive compounds including polyphenols (e.g., flavonoids and non-flavonoids), which have been shown to prevent oxidative processes. These compounds confer to fruits a significant antioxidant capacity related to numerous healthy properties [35].

Based on this, Small Sensor System (S3) analysis was able to follow step by step the wine vinegar production, showing how it was possible to discriminate each phase regarding the volatile compounds produced. Therefore, during the microbial evolution promoted by the yeasts, volatile compounds were released, causing a gradual evolution of the volatiloma detected by the array of sensors utilized in this project. As a matter of fact, Núñez-Carmona et al. [26] affirmed how microorganisms’ metabolic activity causes a slow release of VOCs contained in the analyzed matrix. Consequently, it is possible to consider the volatile compounds as markers.

### Data Processing

**Figure 5:**
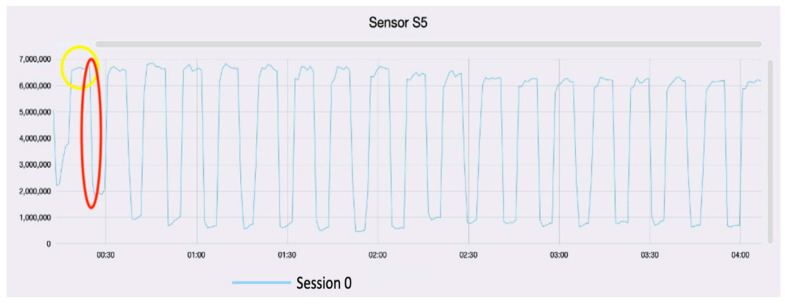
Graph model of sensor response to sample vinegar. *x*-axis presents the time of the analysis, *y*-axis presents the electrical resistance (Ohm). The baseline is the steady line circled in yellow. The sampling is the huge fall circled in red.

The first approach is the visualization of the graph to validate the analysis. The trend of each sampling must be standardized and has a defined pattern. Figure 5 shows the response graph of vinegar analysis. Generally, the more intense the volatiloma the deeper is the fall of the signal.

As a matter of fact, volatile fingerprint intensity is a significant index to reflect the quality of vinegar. During acetic fermentation, the quantity of the compounds often regularly changes as fermentation time, such as ethanol, acetic acid, fatty acids, acetates, and some VOCs [36]. For example, the ethyl esters decrease rapidly at the start of the process and then remain almost constant, together with the pH value. In addition, most VOCs varies rapidly at the start and then become slight and eventually might reach equilibrium during the acetic fermentation [37].

**Figure 6:**
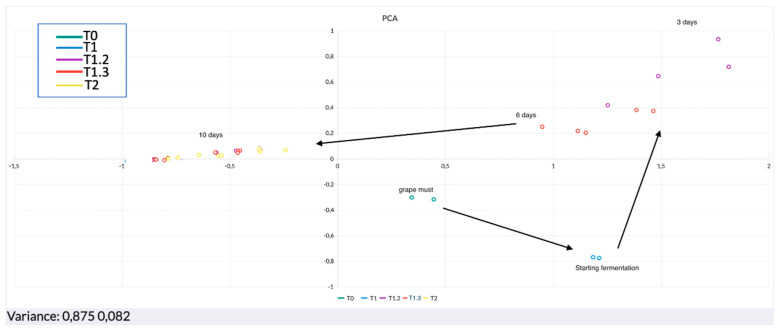
PCA alcoholic fermentation.

**Figure 7:**
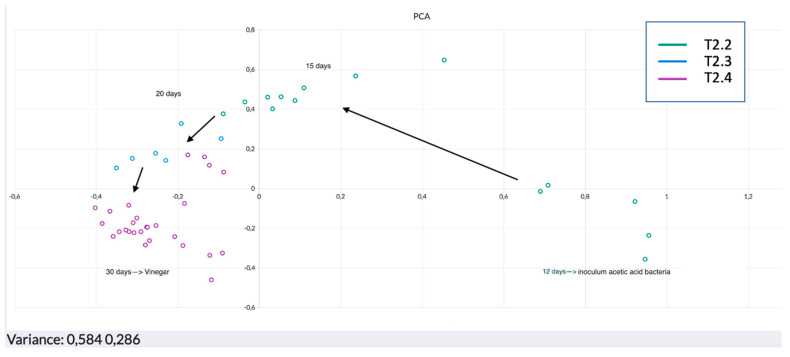
PCA acetic fermentation.

Two different PCAs are shown in Figure 6 and Figure 7, illustrating the alcoholic (from T0 to T2) and the acetic (from T2.2 to T2.5) fermentation phases, respectively.

It is possible to say that overall PCA showed the explained variance (EV) as never under 86%. This represents an optimum result since at least 86% of the total variability of the samples was enclosed between the hyperplane (enclosed between the first two principal components). PC1 is always the component with a larger load, reaching the values of 87.5% and 58.4% in the first (Figure 6) and second PCA (Figure 7), respectively. A closer look at Figure 6 shows the first steps of production. It is possible to follow the production from the must (T0). Once the pied de cuve was done, some evolution of the volatile fraction was immediately noticed by the sensors, as it is clear from the position on the hyperplane of the replicates about the beginning of the fermentation (T1). From this moment, the microbiological growth will strongly affect the volatiloma releasing metabolites that will enrich the volatile fraction. As a matter of fact, the analysis from the third day (T1.2) detected a clear difference as the samples are perfectly discriminated from the previous (starting fermentation). As a consequence, the evolution continued in parallel with the microbial flora changing, up to the end of the first phase, the alcoholic fermentation. The trend of the response between time T1.3 and T2 shows the gradual transition between the intermediate period of alcoholic fermentation and the end of fermentation itself. After 10 days (T2), there is a steady period when the wine is formed. On the twelfth day, a starter culture of *Acetobacter aceti* was inoculated in order to start the acetic fermentation. This phase can be followed from the PCA in Figure 7. In general, a change in the concentration of the major VOCs takes place during the acetic fermentation process. In particular, ethanol drops to ≤1%, while acetic acid increases from values of ≤0.02% to ≥10% [38,39]. After only 3 days a consistent difference was detected by the sensors. This is clearly noticeable as the replicates after 15 days clustered in the opposite side of the hyperplane. Overall, the movements of the samples on the hyperplane stopped at day 30, the moment when it was detected a stability regarding the volatile fraction of the product and when the replicates concentrated in an area. This time would identify the correct formation of the vinegar.

## 4. Conclusions

In conclusion, a new kind of innovative approach was tested to follow step by step the production of vinegar. The real time monitoring of the food supply chain is a subject of great interest and currently highly investigated. The evolution of wine vinegar was analyzed continuously from the grape to the vinegar and up to the beginning of the aging process. Each step was perfectly recognized due to the metabolic release promoted by the microbial evolution of yeasts. The analyses were performed through a new type of chemical semiconductor metal oxide (MOX) gas sensor, which is primarily responsible for the analysis of the response. The device used for the analysis is named—Small Sensor Systems—S3+ developed by Nano Sensor Systems S.r.l., and it is able to detect volatile compounds that are transported by a pump inside the device from samples. The system allows for the collection and analysis of the data acquired in the cloud, making S3+ an IoT device for the management and control of signals. The sensor response is based on the change of its electrical resistance [40] over time caused by interactions with different kinds of volatile compounds or surrounding environments. The data analysis was processed by statistical multivariate analysis of principal component analysis (PCA). Based on this, the gas sensors were able to classify the volatiloma at different alcoholic and acetic fermentation stages, respectively. This would give the possibility to the producer to automatically select the moment when the product is ready and mature.

## Data Availability

Not applicable.
